# Epstein-Barr Virus BART9 miRNA Modulates LMP1 Levels and Affects Growth Rate of Nasal NK T Cell Lymphomas

**DOI:** 10.1371/journal.pone.0027271

**Published:** 2011-11-10

**Authors:** Rajesh Ramakrishnan, Hart Donahue, David Garcia, Jie Tan, Norio Shimizu, Andrew P. Rice, Paul D. Ling

**Affiliations:** 1 Department of Molecular Virology & Microbiology, Baylor College of Medicine, Houston, Texas, United States of America; 2 Department of Virology, Division of Virology & Immunology, Medical Research Institute, Tokyo Medical and Dental University, Tokyo, Japan; Karolinska Institutet, Sweden

## Abstract

Nasal NK/T cell lymphomas (NKTCL) are a subset of aggressive Epstein-Barr virus (EBV)-associated non-Hodgkin's lymphomas. The role of EBV in pathogenesis of NKTCL is not clear. Intriguingly, EBV encodes more than 40 microRNAs (miRNA) that are differentially expressed and largely conserved in lymphocryptoviruses. While miRNAs play a critical role in the pathogenesis of cancer, especially lymphomas, the expression and function of EBV transcribed miRNAs in NKTCL are not known. To examine the role of EBV miRNAs in NKTCL, we used microarray profiling and qRT-PCR to identify and validate expression of viral miRNAs in SNK6 and SNT16 cells, which are two independently derived NKTCL cell lines that maintain the type II EBV latency program. All EBV BART miRNAs except BHRF-derived miRNAs were expressed and some of these miRNAs are expressed at higher levels than in nasopharyngeal carcinomas. Modulating the expression of BART9 with antisense RNAs consistently reduced SNK6 and SNT16 proliferation, while antisense RNAs to BARTs-7 and -17-5p affected proliferation only in SNK6 cells. Furthermore, the EBV LMP-1 oncoprotein and transcript levels were repressed when an inhibitor of BART9 miRNA was transfected into SNK6 cells, and overexpression of BART9 miRNA increased LMP-1 protein and mRNA expression. Our data indicate that BART9 is involved in NKTCL proliferation, and one of its mechanisms of action appears to be regulating LMP-1 levels. Our findings may have direct application for improving NKTCL diagnosis and for developing possible novel treatment approaches for this tumor, for which current chemotherapeutic drugs have limited effectiveness.

## Introduction

EBV is a member of the herpes virus family and is a pre-eminent human oncogenic virus with a causal relationship to several malignancies, including endemic Burkitt's lymphoma (eBL), nasopharyngeal carcinoma (NPC), a proportion of gastric carcinomas (GC), NKT-cell lymphomas (NKTCL), Hodgkin disease (HD), post-transplant lymphoma-like disease (PTLD), and leiomyosarcomas [1], [2]. Within the context of AIDS, EBV is associated with a proportion of non-Hodgkin lymphomas, almost all HD, and leiomyosarcomas. The EBV genome contains over 170,000 bp encoding more than 80 genes. EBV gene expression during latency and tumorgenesis consists of distinct combinations of six nuclear proteins (EBNAs), three membrane proteins (LMPs) and multiple noncoding RNAs, including over 40 miRNAs [3], [4], [5], [6], [7]. While the EBV latent proteins have been investigated intensively for some time, the contribution of EBV-encoded miRNAs or altered cellular miRNA expression in EBV-induced cancers has not been fully explored.

The EBV miRNAs were the first viral encoded miRNAs discovered [6]. MiRNAs are ∼22 nt transcripts that form imperfect duplexes with target mRNAs and thereby inhibit their expression. MiRNAs typically target the 3′ UTR of mRNAs and the average magnitude of repression of the encoded protein is ∼30% [8]. EBV miRNAs are primarily derived from a group of alternatively spiced RNAs transcribed from the BamH1A region of the genome (BamA rightward transcripts or BARTs) [3], [4], [5], [6]. The BARTs encode a large number of miRNAs and with the exception of mir-BART2; the majority are derived from two clusters. A cluster of 3 miRNAs has also been identified which are derived from the BHRF1 gene. The sum total of at least 40 EBV-encoded miRNAs dramatically increases the complexity of potentially biologically active molecules encoded by EBV during latent infection [9]. Like many of the miRNAs discovered to date, the functions of the EBV-encoded miRNAs remain poorly understood. It has been hypothesized that herpesvirus miRNAs, including those encoded by EBV, Cytomegalovirus (CMV), and Kaposi's sarcoma- associated Herpesvirus (KHSV), may facilitate the viral life cycle by blocking innate or adaptive immune responses or by interfering with the appropriate regulation of apoptosis, cell growth, or DNA replication in infected cells [9]. Herpesvirus miRNAs might also target mRNAs for viral genes that regulate the productive lytic cycle, thus having a role in maintaining latency or modulating productive lytic infection. EBV-encoded miRNAs can target both viral and cellular genes. EBV mir-BART2 targets the EBV DNA polymerase mRNA for degradation [10], which inhibits lytic replication and miRNAs from BART cluster 1 may target the viral LMP-1 protein [11]. In addition, mir-BART5 targets the pro-apoptotic factor PUMA and mir-BHRF1-3 targets the chemokine/T-cell attractant CXCL11 [12], [13]. Dysregulation of cellular miRNAs following B cell infection has also been described [11], [14], [15], [16], [17]. The cellular miRNAs 146a and 155 regulate lymphocyte signaling and gene expression pathways in this context.

Three general patterns of viral gene expression have been identified in EBV-associated cancers [1], [2]. Latency I is characterized by expression of EBNA-1, while latency II is characterized by expression of EBNA-1 along with LMP1 and 2. Latency III is characterized by expression of all EBNAs and LMPs and is typically associated with B cells infected with EBV in vitro or in lymphomas in the immunosuppressed. EBV miRNAs are expressed in all EBV infected tumor cells, although they are differentially expressed in some tumors [3], [4], [5], [6], [7], [18], [19], [20], [21]. The context in which miRNA functions are investigated may be particularly important since the ubiquitous and powerful activities of all the latent proteins expressed in latency III could mask some of the activities contributed by miRNAs. Recently, several cell lines have been isolated from EBV-associated NKT cell lymphomas, which appear to select for latency II in both primary tumor tissues as well as the cell lines [22], [23]. Thus, NKTCL cell lines may be a powerful model system to investigate the functions of EBV gene products within the context of latency II and may lead to insights into miRNA functions in EBV-associated HD, GC, and NPC, for which few practical cell culture systems are available.

Nasal NK/T cell lymphomas (NKTCL) are a heterogeneous group of tumors, so named because some tumors have an NK phenotype (CD3^−^, CD56^+^) and some have a T cell phenotype (usually CD4^+^/CD3^+^, but sometimes CD8^+^ CD3^+^ and sometimes CD3^+^ 4^−^, 8^−^ gamma delta) [24], [25]. NKTCL is a distinct clinical entity characterized by necrotic lesions in the nasal cavity, nasopharynx, and palate. These are generally aggressive tumors with poor prognosis [24], [25]. A universal feature of these tumors is the consistent and strong association with EBV, although the precise role of the virus in this disease remains poorly understood. Analysis of primary tumor tissue has shown a latency II pattern of EBV gene expression [22], [23]. At least 7 cell lines of both NK and T cell-like phenotypes have been derived from primary tumors. These include NK-like (CD3^−^, CD56^+^) SNK1, -6, -10 and T-cell-like (CD3^+^, CD56^+^, TCRγ/δ^+^) SNT 8, -13, 15, -16 cell lines [22], [23]. The cell lines, like the primary tumor tissues from which they were derived, retain latency II EBV expression patterns and the EBV genome is clonal. EBV expresses more than 40 miRNAs, but which ones are expressed in NKTCL remains unknown. We hypothesized that specific viral and cellular miRNAs are likely to play a role in the genesis and maintenance of NKTCL. To address this, we utilized microarrays and quantitative PCR to identify EBV miRNAs that are expressed in established NKTCL cell lines. Transfection of antisense oligonucleotides to some of the abundantly expressed EBV miRNAs revealed that at least one of them, BART9, contributes significantly to NKTCL proliferation. The results provide new information about the expression pattern of EBV encoded miRNAs in NKTCLs and identified a novel function for the EBV-encoded BART9 miRNA.

## Results

### NKTCL stably maintain the EBV Type II latency program

**Figure 1:**
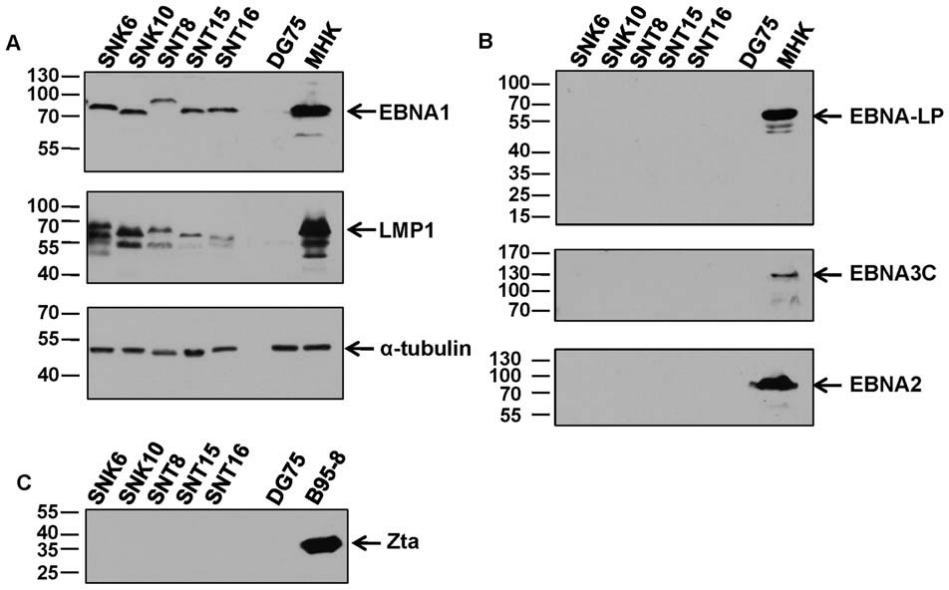
Characterization of NK T cell lymphoma cell lines by immunoblot analysis. Cell lysates were prepared from two NK-like (SNK6, SNK10) and three T cell-like (SNT8, SNT15 and SNT16) NKTCLs. Immunoblots were performed to analyze the expression of the indicated EBV latent and lytic proteins. EBV negative DG75 cells were used as a negative control and EBV positive MHK cells, which maintain Latency III gene expression and express all of the latent proteins, were used as a positive control.

In tumors, EBV displays latency programs characterized by specific patterns of viral gene expression. In Burkitt's lymphoma, Type I latency is seen while Type II latency is observed in nasopharyngeal carcinoma, gastric carcinoma, and Hodgkin's disease. Type III latency is often restricted to B lymphomas in immunodeficient patients [26]. Although previous studies have found that NKTCLs have a type II latency phenotype, it is common for some EBV positive cell lines to drift towards type III latency in culture. To confirm the latency phenotype under our culture conditions, we tested five NKTCL cell lines for latent and lytic gene expression. We found that the two SNK (SNK6 and SNK10) and three SNT (SNT8, SNT15 and SNT16) cell lines expressed EBNA1 and LMP1 (Figure 1A). These cell lines did not express the other EBNA proteins, EBNA-LP, EBNA3C and EBNA2 (Figure 1B). We also found that there was no expression of Zta lytic protein (Figure 1C). These data indicate that the NKTCL cell lines stably exhibit a Type II latency program.

### miRNA microarray profiling of NKTCL

Nasal NK/T cell lymphomas (NKTCLs) have been demonstrated to be consistently associated with Epstein-Barr virus (EBV) as all cases are EBV positive [27], [28], [29]. EBV encodes at least 40 microRNAs (miRNA) [3] and there is increasing evidence for the role that miRNAs play in malignant transformation of cells [30]. Therefore, to investigate the role of EBV miRNAs in NKTCL oncogenesis, we first carried out miRNA microarray profiling. We isolated total RNA from two representative NKTCL cell lines, SNK6 and SNT16. Both SNK6 and SNT16 express cellular and EBV proteins that are consistent with prototypical NK and T-cell like NKTCLs respectively. These cell lines were also chosen for microarray profiling and further analysis because of their robust growth and viability in cell culture relative to other known SNK or SNT cell lines. A miRHumanVirus microarray chip was used to examine the expression levels of 1100 mature miRNAs that included human (875) and viral (225) miRNAs. The probes also included 44 EBV miRNAs in the microarray chip.

**Figure 2:**
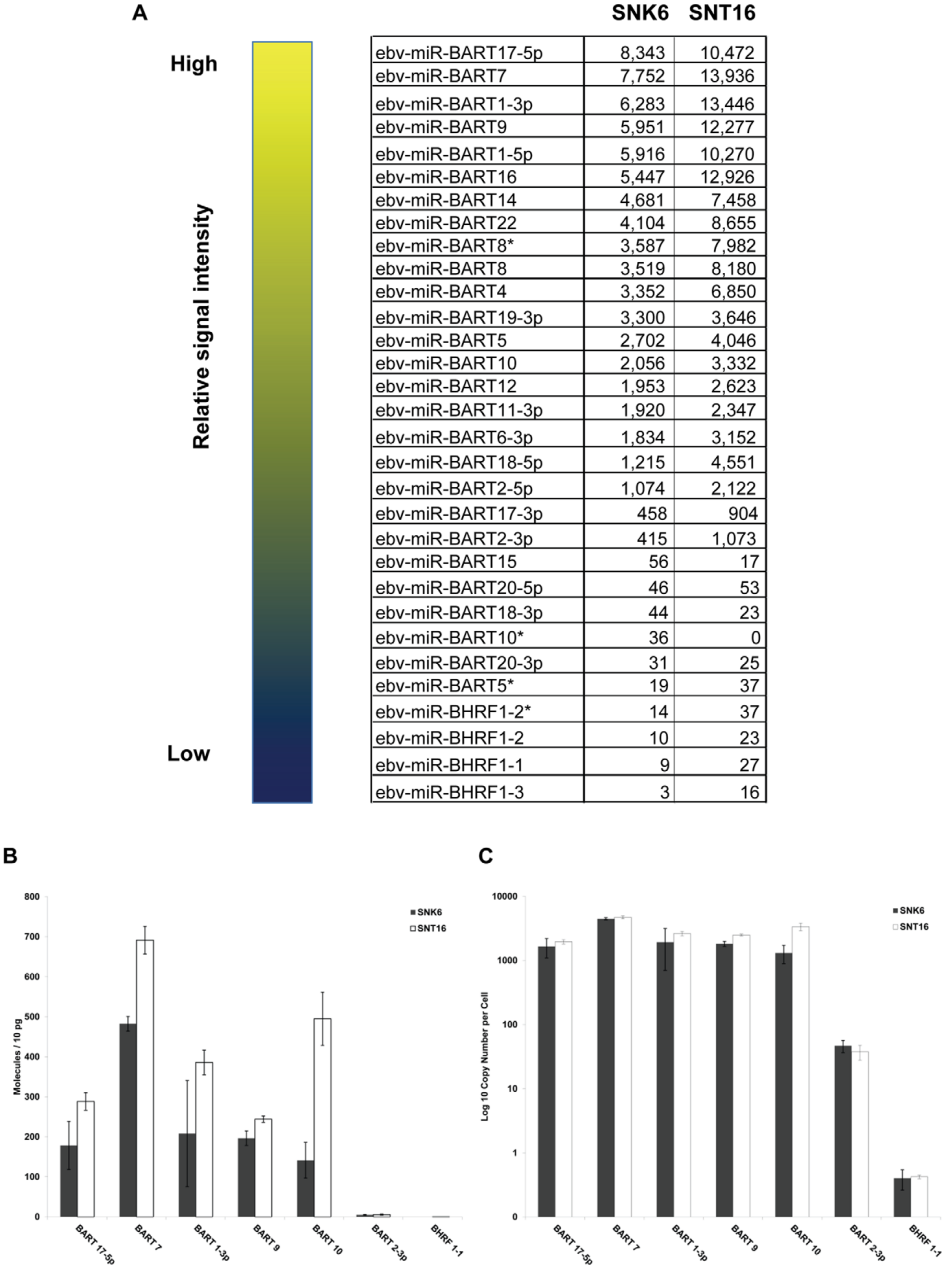
MiRNA expression profile in NK T cell lymphoma cell lines. (**A**) The table shows the median expression values from normalized, log-ratio (base 2) data sets of representative EBV miRNAs in SNK6 and SNT16 cell lines arranged in a descending order of expression levels. (**B**) Taqman qPCR for selected EBV miRNAs in SNK6 and SNT16 cells. The indicated EBV miRNAs were quantified using a stem-loop PCR protocol described by Chen et al [46] for detecting miRNAs. The copy number of each of the miRNAs was determined by reverse transcription and amplification of synthetic miRNAs. The graph represents miRNA expression as molecules per 10 picogram RNA. (**C**) Taqman qPCR for indicated EBV miRNAs in SNK6 and SNT16 cells as described in (B). The data are presented as copy number of EBV miRNA per cell.

We used the criteria and statistical parameters described in the Methods to analyze the EBV miRNA expression patterns in the two NKTCL cell lines. Using a median expression value cut-off of 500, we identified 19–21 EBV miRNAs that were present at relatively high levels in SNK6 and SNT16 cell lines (Fig. 2A). To verify the reliability of the microarray data, we selected seven EBV miRNAs whose expression in the microarray ranged from high to low and carried out Taqman PCR on total RNA extracted from SNK6 and SNT16 cells. To more easily compare the relative expression levels of these miRNAs to previous studies, miRNA levels are shown normalized to either 10 pg total RNA or as copy numbers per cell. As shown in Figure 2B and C, the relative expression level of EBV miRNAs BART17-5p, BART7, BART1-3p, BART9, and BART10 was at least one log higher than EBV miRNA BART2-3p in both SNK6 and SNT16 cells. The levels of the miRNAs were also higher in SNT16 cells than SNK6 cells. This is in agreement with the microarray data which also showed a higher expression level of the selected miRNAs in SNT16 cells compared to SNK6 cells (Fig. 2B). Notably, BHRF1 derived miRNAs were nearly undetectable (Fig. 2A–C). These data indicate that the microarray profiling data are generally reliable and this analysis has therefore determined the set of EBV miRNAs which are expressed in the SNK6 and SNT16 cell lines.

### Reducing EBV miRNA levels affect SNK6 and SNT16 growth rate

**Figure 3:**
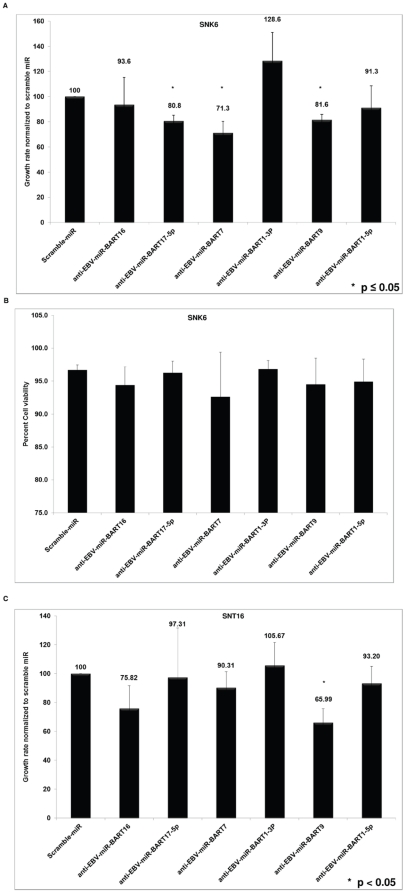
Inhibiting EBV BART miRNA levels affect NKTCL growth rate without affecting cell viability. (**A**) SNK6 cells were transfected with antisense to the indicated EBV miRNAs and cell numbers counted every 24 hours for three days. Cell growth rate was calculated as difference in cell numbers between the 24 hour and 72 hour time point and compared with cells transfected with control Scramble miRNA. Data shown are the average ± SD from three independent experiments. (* represents p value of ≤0.05 in a paired t-test). (**B**) SNK6 transfected with antisense EBV miRNAs were analyzed for viability by Trypan blue exclusion in a Vi-CELL counter every 24 hours for three days. The data presented is the cell viability at 72 hours post-transfection and is the average ± SD from three independent experiments. (**C**) SNT16 cells were transfected with antisense to the indicated EBV miRNAs and cell growth rate analyzed as described above. Data shown are the average ± SD from three independent experiments. (* represents p value of <0.05 in a paired t-test).

miRNAs regulate many genes including, those involved in cell growth [31]. We first investigated the consequences of blocking EBV miRNA function on the growth rate of SNK6 and SNT16 NKT-cell lines. Based on the miRNA microarray profile (Fig. 2), we chose six EBV miRNAs that were expressed at high levels in both cell lines. The six EBV miRNAs were individually inhibited by transfection LNA-modified antisense oligonucleotides. Samples were collected every 24 hours for three days and cell numbers and viability analyzed. The anti-EBV-miR-BART9, anti-EBV-miR-BART7 and anti-miR- BART17-5p showed a statistically significant reduction in SNK6 cell growth (∼19%, ∼20% and ∼29%, respectively) (Fig. 3A). EBV-miR-BART1-5p and EBV-miR-BART16 antisense oligonucleotides did not have statistically significant effects on SNK6 growth rate. Also there was no significant effect on the viability of the SNK6 cells upon inhibition of any of the EBV miRNAs shown in Figure 3A (Fig. 3B). In SNT16 cells, only anti-EBV-miR-BART9 showed a statistically significant decrease (∼34%) in cell growth. Anti-EBV-miR- BART16, anti-EBV-miR-BART17-5p, anti-EBV-miR-BART7, anti-EBV-miR1-5p affected cell growth by ∼25%, ∼3%, ∼10% and ∼7%, respectively, but these differences were not statistically significant in a paired t-test (Fig. 3C). We noted that there was a decrease of ∼20% in viability of SNT16 cells when the levels of EBV miRNAs were reduced (data not shown). Anti-EBV-miR-BART1-3p showed an increase in SNK6 and SNT16 cell growth, but this difference was not statistically significant (Fig. 3A and C). A scrambled control miRNA had no detectable effect on proliferation or viability on either cell line (Fig. 3). This data suggests that the expression levels of some EBV miRNAs may play a role in cell proliferation.

### Inhibiting EBV miR-BART9 reduces LMP1 protein and mRNA expression in SNK6 cells

**Figure 4:**
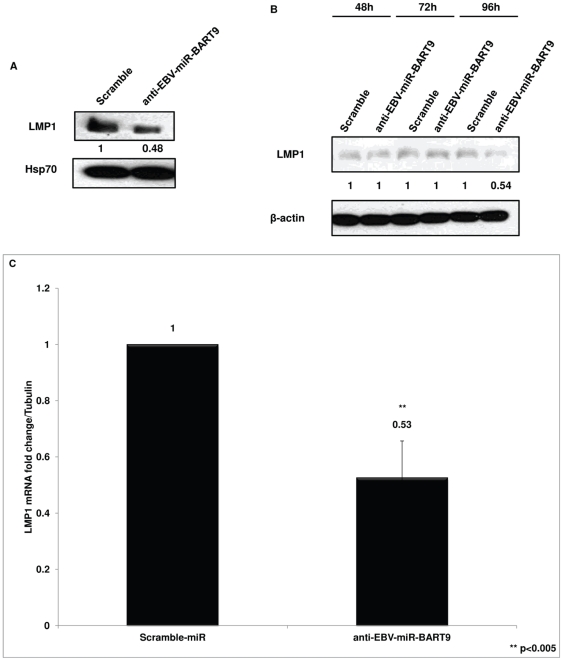
Immunoblot and Q-RT-PCR analysis of LMP1 expression in SNK6 following inhibition of EBV-BART9 miRNA. (**A**) SNK6 cells were transfected with anti-EBV-BART9 miRNA or Scramble control miRNA and cell lysates prepared 96 hours post-transfection. LMP1 protein expression was analyzed in immunoblots. When compared to cells transfected with control miRNA and normalized to β-actin loading control, quantification of immunoblots showed that BART9 inhibition reduced LMP1 protein levels by ∼50%. (**B**) SNK6 cells were transfected with control or anti-EBV-BART9 miRNA and samples collected every 24 hours in a time-course experiment. Cell lysates were prepared and immunoblot analysis carried out to determine LMP1 expression. Quantification of LMP1 levels using Image J as described above showed that LMP1 protein levels are reduced only at later time-point. (**C**) SNK6 cells were transfected with either anti-EBV-BART9 or control miRNA and cells collected 96 hours post-transfection. Total RNA was extracted and cDNA synthesized using iScript cDNA synthesis kit. Using LMP1 specific primers, Q-PCR was carried out and data analyzed using the ΔΔCt method. Data shown is the average ± SD from three independent experiments. (** represents p value of <0.005 in a paired t-test).

Because reducing BART9 levels affected growth rate in SNK6 and SNT16 cells (Fig. 3), we focused further experiments on the BART9 miRNA. EBV-encoded LMP1 triggers multiple cellular signaling pathways that influence cell growth [32]. We carried out immunoblot analysis to investigate if the effect on growth rate upon reduction of EBV BART miRNA was a result of altered LMP1 expression. SNK6 cells were transfected with either control miRNA (Scramble) or anti-EBV-miR BART9 and cells were lysed 96 hours post-transfection. Immunoblots were performed and probed for LMP1 levels. We found that inhibiting EBV miR-BART9 reduced LMP1 protein expression by almost 50% when normalized to the Hsp70 loading control and relative to the control miRNA (Fig. 4A). In these experiments, we also probed for lytic protein Zta in order to examine if the activation of EBV lytic program was the reason for reduced SNK6 cell growth rate and found no detectable expression of Zta protein (data not shown). We also investigated the kinetics of anti-EBV-miR-BART9 effect on LMP1 level by carrying out a time-course experiment. We observed that there was a ∼46% reduction of LMP1 protein level 96 hours post-transfection of anti-EBV-miR-BART9 compared to control miRNA (Fig. 4B).

We next examined if the ∼50% reduction in LMP1 protein level following inhibition of BART9 was a consequence of reduced LMP1 mRNA expression. SNK6 cells were transfected with anti-EBV-miR-BART9 and total RNA extracted from the cells 96 hours post-transfection. cDNA was synthesized and Q-PCR was performed using LMP1 specific primers. We found that there was ∼2-fold decrease in the LMP1 mRNA levels when BART9 was inhibited (Fig. 4C). This data suggests that BART9 miRNA functions as a positive factor for LMP1 at the level of mRNA accumulation.

### EBV miR-BART9 has a positive effect on LMP1 protein and mRNA expression in SNK6 cells

**Figure 5:**
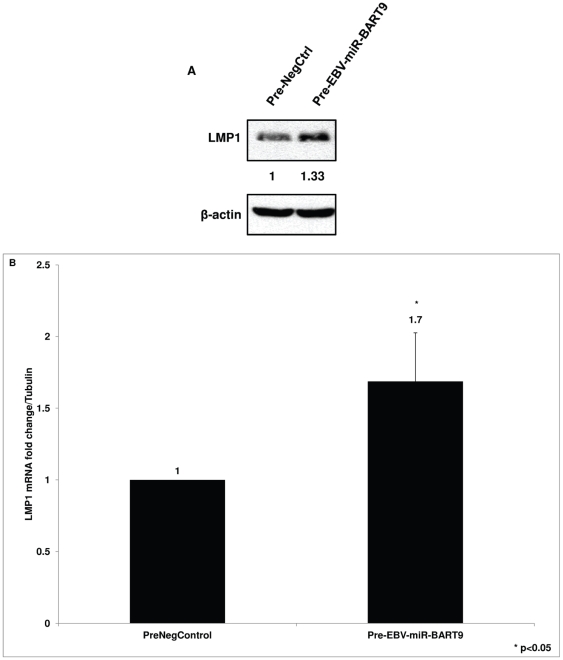
Precursor EBV-BART9 miRNA increases LMP1 protein and mRNA levels in SNK6 cells. (**A**) SNK6 cells were transfected with precursor EBV-BART9 or control miRNA. The cells were collected 96 hours post-transfection and cell lysates prepared for immunoblot analysis. Quantification of immunoblots showed a ∼33% increase in LMP1 protein levels in cells transfected with EBV miRNA compared to control miRNA transfected cells when normalized to loading control. Data shown is a representative immunoblot from three independent experiments. (**B**) Total RNA was extracted from SNK6 cells transfected with precursor EBV-BART9 or control miRNA. Following cDNA synthesis, LMP1 mRNA levels were analyzed by Q-RT-PCR. Data presented is the average ± SD from three independent experiments. (* represents p value of <0.05 in a paired t-test).

If BART9 does indeed have a positive influence on LMP1 expression, then increasing its level might be predicted to increase LMP1 expression, in contrast to the effect of the antisense BART9 miRNA (Fig. 3, 4). To test this prediction, we transfected a precursor for miRNA-BART9 (pre-EBV-miR-BART9) into SNK6 cells and performed immunoblots and Q-RT-PCR to examine levels of LMP1 protein and mRNA, respectively. We found that increasing BART9 levels increased LMP1 protein expression by ∼33% relative to cells transfected with the precursor negative control miRNA (pre-NegCtrl) when normalized to the loading control β-actin (Fig. 5A). We also found that over expressing BART9 increased the LMP1 mRNA level by a factor of 1.7 (Fig. 5B).

### Increase in EBV-miRNA-BART9 level modestly affects SNK6 cell growth

**Figure 6:**
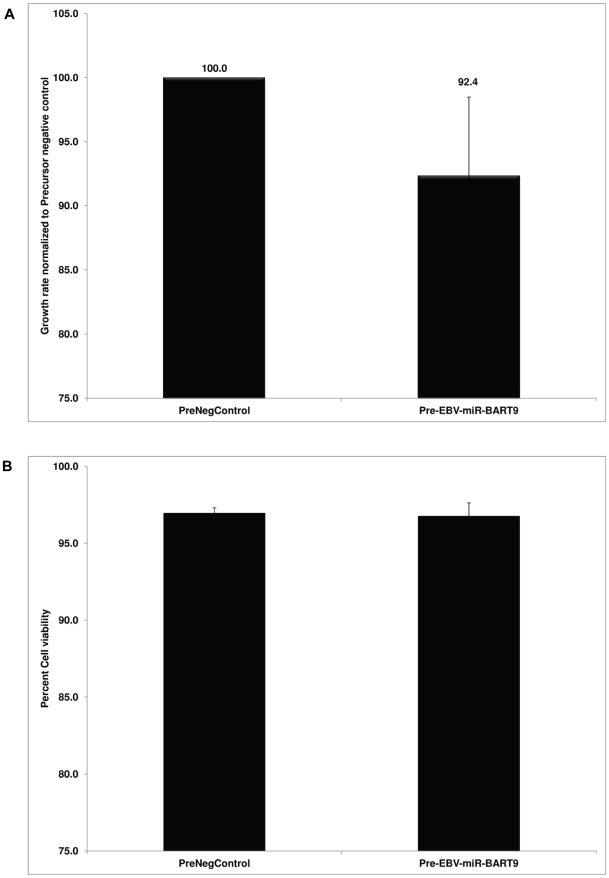
Increasing EBV-BART9 miRNA level has a subtle effect on SNK6 growth rate. (**A**) Precursor EBV-BART9 miRNA or control miRNA were transfected into SNK6 cells and samples collected every 24 hours for three days. Growth rate of SNK6 cells was determined by calculating cell numbers. When normalized to cell numbers in control miRNA transfected cells, there was ∼8% reduction in SNK6 growth rate. The data shown is the average ± SD from three independent experiments. (**B**) In the experiments described above, SNK6 were analyzed for viability by Trypan blue exclusion in a Vi-CELL counter at every time point. The data shown is the cell viability at 72 hours post-transfection and is the average ± SD from three independent experiments.

The role of LMP1as the oncoprotein of EBV is dependent on its expression level. While LMP1 has been reported to promote cellular transformation, increased expression of LMP1 can inhibit cell growth [33]. We next investigated whether increasing BART9 levels would inhibit SNK6 cell growth. Precursor BART9 was transfected into SNK6 cells and samples were collected every 24 hours for three days and cell numbers and viability analyzed. We found that increasing BART 9 levels modestly (∼8%) affected SNK6 cell growth rate (Figure 6A) without affecting viability (Figure 6B). Although the data for reduction in growth rate of SNK6 following over-expression of BART9 did not show a statistically significant difference in a paired t-test, the results of three independent experiments showed a clear and reproducible trend of reduced growth. This suggests that the level of LMP1 needs to be regulated stringently as an increase (Figure 6) or decrease below a threshold point (Figure 3 and 4) has an inhibitory effect on SNK6 cell growth. To determine whether the effects of BART9 miRNA on LMP1 expression are directed through the 3′UTR of the LMP1 mRNA, we cotransfected a BART 9 miRNA precursor with an LMP1 expression plasmid containing the natural 3′UTR and a plasmid lacking this element in HeLa cells. Under these conditions, BART9 had no effect on LMP1 expression from either expression plasmid, suggesting that the effects of BART9 on LMP1 expression are indirect (data not shown).

## Discussion

In this study we show that ∼20 EBV miRNAs are abundantly expressed in Nasal NK/T cell lymphomas (NKTCL). We also provide evidence that modulating EBV miRNA levels impacts NKTCL growth rate. Furthermore, we found a direct correlation between levels of EBV-miR-BART9 and LMP1 protein and mRNA expression. Together, these observations suggest that BART9 miRNA positively modulates expression of LMP1 and one manifestation of perturbing this regulation is a retardation of NKTCL cell growth.

A number of studies have characterized EBV miRNA expression and their roles in nasopharyngeal carcinomas [4], [11], [19]. Other studies have focused on the role of cellular miRNAs in NKTCLs [34] and other human cancers [35]. While EBV miRNAs have been found to be conserved evolutionarily [3], they are differentially expressed in different cell types [3], [7]. However, to our knowledge this is the first study to determine the expression of EBV miRNAs in NKTCLs. We found that at least 19 EBV miRNAs are abundantly expressed in NKTCLs. Indeed, these 19 miRNAs appear to be expressed at levels 2–3 logs higher than their expression in NPCs [19]. We note that a limitation of our study is that only two NKTCL cell lines and no primary tumors were profiled. Nevertheless, our data suggest that even though EBV viral gene expression might be similar to NPC and Hodgkin disease [36], miRNA expression could vary greatly between these two tumors.

What role might these EBV miRNAs play in NKTCLs? Cancer is a disease where cell proliferation is dysregulated. A number of studies have demonstrated a connection between miRNAs and cellular differentiation and in many instances miRNAs act as oncogenes by down-regulating tumor suppressors [30], [37]. In this study, we found that inhibiting EBV miRNAs slowed the growth rate of NKTCLs. This reduction in proliferation was not because of loss of cell viability. These EBV miRNAs may be functionally analogous to cellular miRNAs like miR- 106b that targets the cell cycle inhibitor p21^Cip1^ [38] or miR-221 and miR-222 that regulate p27^kip1^ [39]. Since EBV miRNAs are evolutionarily conserved [3], it is also possible that they target viral proteins as is the case with EBV-miR-BART17-5p that has been reported to regulate LMP1 [11] or EBV-miR-BART2 that down-regulates BALF5 viral DNA polymerase [10]. In the SNK6 cell line we observed that inhibiting EBV-miR-BART9 reduced the level of LMP1 mRNA and protein. Furthermore, over-expression of BART9 miRNA resulted in increase of LMP1 protein and transcript levels. This suggests that in NKTCLs, BART9 miRNA likely regulates LMP1 mRNA expression and we favor the hypothesis that this is an indirect regulation as a reporter plasmid containing the 3′ UTR of the LMP1 mRNA was not responsive to BART9 in transient expression experiments (data not shown). BART9 may indirectly up-regulate LMP1 by targeting a repressor of its expression. When BART9 is inhibited, the level of this putative repressor may be increased resulting in decrease of LMP1 transcript and protein levels. Alternatively, BART9 may be involved somehow in maintaining LMP1 mRNA stability which has been reported to have a long half-life [40]. In this scenario, BART9 may stabilize LMP1 mRNA, and inhibition of BART9 thus renders the LMP1 mRNA susceptible to degradation.

A tight regulation on the expression of LMP1 is beneficial to EBV and its survival in infected cells. For instance, if LMP1 expression is consistently high, it can either result in cell growth arrest [41], inhibit viral and cellular promoters [42] or enhance epitope presentation to cytotoxic T cells [43]. However, under certain conditions, it might be beneficial for EBV to induce LMP1 expression for a short period of time. Notably, a recent study reported the transient upregulation of LMP1 by the p38 signaling pathway [44].

In summary, we have shown that 19 EBV miRNAs are abundantly expressed in NKTCLs and their levels are likely to be important in maintaining cell growth. Our data also indicate that EBV BART9 is involved in regulating LMP-1 expression in these cells. This has implications in mechanisms of lymphomagenesis and future experiments could be directed at investigating the role of EBV miRNAs and its regulation of cellular targets.

## Methods

### Cell lines

The NK-T cell lymphoma (NKTCL) cell lines, SNK6, SNK10, SNT8, SNT15, SNT16 were obtained from Norio Shimizu (Tokyo Medical and Dental University). The cells were cultured in RPMI1640 supplemented with 10% fetal bovine serum, 1% Penicillin-Streptomycin and 250 ng/ml Fungizone (Amphotericin B; Invitrogen) and 600 IU of IL-2.

### MiRNA microarray analysis and validation

Total RNA was isolated from SNK6 and SNT16 cells using the miRNAeasy kit (Qiagen) as per manufacturer's protocol. RNA was analyzed by LC Sciences (Houston, TX) with miRNA microarrays using the μParaflo microfluidic chip technology and all data is MIAME compliant. The detailed process can be found at http://www.lcsciences.com. Briefly, photogenerated reagent chemistry probes for miRNAs were *in situ* synthesized on chips with three repeats for each probe to allow for statistical analysis. MiRHumanViruses version 13 arrays were used to detect a total of 1100 unique mature miRNAs comprising of 875 human miRNAs and 225 virus miRNAs. The virus miRNAs included 44 EBV miRNAs. RNA samples from SNK6 and SNT16 cells were labeled with Cy3 for hybridization. The chips included 50 control probes based on Sanger miRBase Release 13 with four-sixteen repeats. The control probes were used for quality controls of chip production, sample labeling and assay conditions. Included in the control probes were PUC2PM-20B and PUC2MM-20B which are the perfect match and single-base match detection probes, respectively, of a 20-mer RNA positive control sequence that is spiked into the RNA samples before labeling. For a transcript to be listed as detectable three conditions had to be met: (1) signal intensity had to be greater than three times background standard deviation; (2) spot co-variance (CV), defined as ratio of standard deviation over signal intensity had to be less than 0.5; (3) the signals from at least 50% of the repeating probes had to be above detection level. Data was normalized using a cyclic LOWESS (Locally-weighted Regression) method [45] to remove system related variations such as sample amount variations, dye labeling bias, and signal gain differences between scanners to reveal biological relevant variations. A t-test was performed on the signals obtained for the repeating probes and p-value calculated. MiRNAs were defined as differentially expressed if they had a p-value<0.01. Clustering analysis was performed with a hierarchical method using average linkage and Euclidean distance metric. The clustering data was represented as a heat map using TIGR MeV (Multiple Experimental Viewer; The Institute for Genomic Research). The microarray data has been deposited in GEO database with accession number GSE30695.

### Validation of miRNA microarray

Selected EBV miRNAs were quantified using a PCR protocol described by Chen *et al* [46] for detecting miRNAs. Briefly, stem-loop primers complementary to specific EBV miRNAs were designed as described by Cosmopoulos *et al* [19]. For each miRNA assayed, 100 ng of total RNA was reverse transcribed using a TaqMan MicroRNA RT kit as described by the manufacturer and a specific stem-loop primer at a final concentration of 50 nM. RNA was prepared using an RNeasy kit (Qiagen) from exponentially growing tissues culture cells. Each 20 ul PCR reaction contained 1 ul of RT product, 1×TaqMan Universal master mix, 1.5 uM forward primer, 0.7 uM reverse primer, and 0.2 uM probe. The reactions were incubated in a 48-well plate at 95°C for 3 min, followed by 40 cycles of 95°C for 15 s and 60°C for 30 s. The copy number of each of the miRNAs was determined by reverse transcription and amplification of synthetic miRNAs that were identical to the published sequences.

### miRNAs and transfection

The miRCURY locked nucleic acid (LNA) modified antisense oligonucleotides to EBV BART miRNAs (anti-EBV-miR-BART) were purchased from Exiqon. The sequence of the antisense oligonucleotides are as follows: Scramble Negative Control (5′-GTGTAACACGTCTATACGCCCA -3′); anti-EBV-miR-BART9 (5′- ACTACGGGACCCATGAAGTGTTA- 3′); anti-EBV-miR-BART17-5p (5′- CTTGTATGCCTGCGTCCTCTTA-3′); anti-EBV-miR-BART7 (5′- CCCTGGACACTGGACTATGATG-3′); anti-EBV-miR-BART1-3p (5′- GACATAGTGGATAGCGGTGCTA-3′); anti-EBV-miR-BART1-5p (5′- CACAGCACGTCACTTCCACTAAGA-3′); anti-EBV-miR-BART16 (5′FAM- AGAGCACACACCCACTCTATCTAA-3′). Precursor EBV miRNA (pre-EBV-miR) was designed based on the sequence in miRBase sequence database (http://microrna.sanger.ac.uk/sequences). pre-EBV-miR-BART9 (5′- UAACACUUCAUGGGUCCCGUAGU-3′) and precursor Negative control miRNA (pre-NegCtrl) were ordered from Ambion/Applied Biosystems.

SNK6 and SNT 16 cells were seeded at 1×10^6^ cells in 24- well tissue culture plates and transfected with antisense or precursor miRNAs using Oligofectamine or Lipofectamine RNAimax according to the manufacturer's protocol. Transfection efficiency of the miRNAs in SNK6 and SNT 16 cells was determined with FAM labeled EBV- BART16 miRNA and was found to be nearly 98% as determined by flow cytometry (data not shown). Cell viability following transfections was measured by Trypan Blue exclusion and found to be ∼95%.

### RT-real time PCR for EBV mRNAs

Independent transfections of anti-EBV-miR-BART 9 or pre-EBV-miR-BART 9 were performed in SNK6 cells. The controls transfected were either Scramble-miRNA (Exiqon) or Precursor-Negative Control (Pre-NegCtrl) (Applied Biosystems), respectively, as described above. Total RNA was isolated using RNeasy mini kit (Qiagen) and RT-real-time-PCR assays carried out for quantification of LMP1 and α-tubulin levels using the Bio-Rad MyIQ single color detection system. Briefly, 10 ng of cellular RNA was reverse transcribed into cDNA using the iScript cDNA synthesis kit (Bio-Rad) in a 20 µl reaction using the manufacturer's protocol. Quantitative real-time PCR was performed using 3 µl of the synthesized cDNA and the iQ™ SYBR Green Supermix (Bio-Rad). PCR reactions were carried out in 96-well format using a Bio-Rad iCycler. Analysis was done by the MyIQ software program (Bio-Rad) and the fold-changes were calculated using the ΔΔCt method as previously described [47] with α-Tubulin as the housekeeping gene control. The primer sequences used for LMP1 have been described previously [48] and were LMP1 (forward) - 5′ AGCCCTCCTTGTCCTCTATTCCTT 3′, LMP1 (reverse) - 5′ACCAAGTCGCCAGAGAATCTCCAA 3′. The primers for α-Tubulin were, α-Tubulin (forward) - 5′ CCTGACCACCCACACCACAC 3′, α-Tubulin (reverse) - 5′ TCTGACTGATGAGGCGGTTGAG 3′.

### Cell proliferation functional assay

SNK6 and SNT16 cells were seeded in 96 well plates at 8×10^5^ cells in 100 µl/well and transfected with 100 pmol of indicated anti-EBV-miRNAs or pre-EBV-miRNAs or control Scramble-miRNA or Pre-Neg-Ctrl. In some experiments, the cells were seeded at 1×10^6^ cells/well. After overnight incubation, the cells were transferred into 24 well tissue culture plates. Cells were collected every 24 hours and analyzed for cell number and viability using the Becton-Dickinson Vi-CELL counter at the Baylor College of Medicine Flow Cytometry Core. The cell counter uses trypan blue exclusion to automatically stain and count cells, as well as assay cell size and viability.

### Immunoblotting

Cells following treatment were lysed with EBCD buffer (50 mM Tris-HCl, pH 8.0, 120 mM NaCl, 0.5% NP-40, 5 mM dithiothreitol) containing protease inhibitor cocktail (Sigma). Immunoblotting was performed as described previously [49]. Monoclonal antibodies used in this study include, LMP1 (S12), EBNA-LP (JF186), EBNA3C (A10), and EBNA2 (R3). Other antibodies obtained commercially included EBNA1 (1EB12, Santa Cruz), Zta (Argene), β-Actin (Sigma) and α-tubulin (Sigma). HRP secondary antibodies were obtained from Jackson Immunolaboratories and Western blots were developed using the SuperSignal West pico kit (Thermo Scientific). Immunoblots were quantified using Image J software [50].
